# Chinese older adults’ prior-to-death disability profiles and their correlates

**DOI:** 10.1186/s12877-024-05105-y

**Published:** 2024-06-01

**Authors:** Chuqian Chen, Lingling Yu

**Affiliations:** 1 Department of Medical Humanities, School of Humanities, Southeast University, Nanjing, China; 2 Jiangsu Ageing-Responsive Civilization Think Tank, Nanjing, China; 3 Department of Philosophy and Science, School of Humanities, Southeast University, Nanjing, China

**Keywords:** Prior to death, Disability, Latent profile analysis, Chinese

## Abstract

**Background:**

Disability prior to death complicates end-of-life care. The present study aimed to explore the prior-to-death disability profiles of Chinese older adults, the profiles’ links to end-of-life care arrangements and place of death, and predictors of the profiles.

**Methods:**

In total, data were extracted from the records of 10,529 deceased individuals from the Chinese Longitudinal Healthy Longevity Survey (CLHLS). Latent profile analyses, bivariate analysis, and multivariate logistic regression were applied to identify prior-to-death disability profiles, explore the profiles’ links to end-of-life care arrangements and place of death, and examine predictors in the profiles, respectively.

**Results:**

Three prior-to-death disability profiles, namely, Disabled-Incontinent (37.6%), Disabled-Continent (34.6%), and Independent (27.8%), were identified. Those with the Independent profile were more likely to live alone or with a spouse and receive no care or care only from the spouse before death. Disabled-Continent older adults had a higher chance of dying at home. Being female, not “married and living with a spouse”, suffering from hypertension, diabetes, stroke or cerebrovascular disease (CVD), bronchitis/emphysema/pneumonia, cancer, or dementia, and dying in a later year were associated with more severe prior-to-death disability patterns. Not having public old-age insurance predicted lower chances of having a Disabled-Incontinent profile, and advanced age increased the chance of having a Disabled-Continent profile.

**Conclusions:**

Three prior-to-death disability patterns were identified for Chinese adults aged 65 years and older. These profiles were significantly linked with the end-of-life caregiving arrangements and place of death among older adults. Both demographic information and health status predicted prior-to-death disability profiles.

**Supplementary Information:**

The online version contains supplementary material available at 10.1186/s12877-024-05105-y.

## Background

### Disability in older adults prior to death

Disability is a great challenge for older adults worldwide. Late-life disability has been associated with depressive symptoms [1], lower quality of life [2], interference with social engagement [3], and even increased suicidal behaviors [4]. Very old adults (aged $\ge$ 80) are at increased risk of disability when they are approaching death, and the rate of change is especially rapid within the last 12 months of life [5].

Disability prior to death complicates end-of-life care. A study in the United States revealed that in the last year of life, the incidence of hospice admission among older adults (aged ≥ 70) in a specific month increased by 10% for each additional disability [6]. Interviews have shown that balancing disability support and end-of-life care is difficult even for patients with long-term physical disabilities [7]. Things can only be more challenging when both disability support needs and end-of-life care needs quickly emerge shortly before death. Before individualized care plans are initiated, it is important for researchers, policy makers, and frontline practitioners to understand disability patterns among older adults prior to death, how such patterns influence end-of-life care arrangements and outcomes, and what factors predict such patterns.

In terms of prior-to-death disability patterns, person-centered analytic methods such as latent profile analysis (LPA) and latent classic growth analysis (LCGA) [8] can provide great insights by identifying subpopulations with specific combinations and changing patterns of observed variables [9], respectively. For instance, the LCGA of an older American adult study sample (aged 72–109 years) revealed three heterogeneous trajectories of disability development within the last 10 years of life: “rapid decline” (52%), “moderate decline” (36%), and “frailty” (12%) [10]. In the sample, between-class differences were found in terms of sex, race, and education level. Moreover, five latent trajectories of functional disability, namely, “persistently severe disability” (10.3%), “persistently mild disability” (13.0%), “accelerated disability” (12.6%), “catastrophic disability” (18.8%) and “minimum disability” (45.2%) [11], were identified in a sample of older Japanese adults (aged $\ge$ 65 years) three years prior to death, and self-rated health was found to be a significant predictor of latent class membership.

To our knowledge, no study has explored the cross-sectional distribution rather than longitudinal changing patterns of disability among older adults prior to death, let alone the links of profiles to end-of-life care arrangements and outcomes or predictors of those profiles.

### The Chinese context

China has the largest aging population in the world [12], and chronic diseases have contributed greatly to disability burdens among these older adults [13]. According to a meta-analysis, 26.2% (95% CI: 23.7–28.6%) of Chinese individuals aged 60 years and older suffer from at least one form of disability [14]. Using data from the Chinese Longitudinal Healthy Longevity Survey, one study reported that 85.4% of Chinese adults aged 65 and above were beyond dependent in their last days of life [15]. With the same dataset from the LCGA, three trajectories of activities of daily living, namely, good and stable (66.5%), declining quickly (19.1%), and declining slowly (14.4%), were identified for older adults in their last 16 years of life [16]. In addition to previous findings, describing the cross-sectional disability profiles of Chinese older adults during the end-of-life care period and their predictors would not only promote a theoretical understanding of the status quo of the last days of life among Chinese older adults but also inform system-level policy-making and individual-level practices to enable better support.

Disability severely hinders the ability of Chinese older adults to take care of themselves [17]. In China, the traditional value of filial piety (family support and respect for older adults) is greatly emphasized by the government in promoting home-based care as the cornerstone for facing the needs of the growing aging population [18]. Therefore, disability among older adults significantly increases their need for intergenerational care [19]. Understanding how the disability patterns of Chinese older adults prior to death influence family-based end-of-life care arrangements would help families care for dying relatives. Moreover, due to the cultural belief that “fallen leaves return to the roots”, the majority of Chinese people prefer to die at home [20], choose to go home to die [21], and actually die at home [22]. In this case, exploring how disability profiles prior to death influence the place of death would help us understand not only which subgroups of older adults need more support in their pursuit of a home death but also which disability profiles should receive more attention and consideration in terms of preparation by institutions such as hospitals and nursing homes.

### The present study

The present study aimed to explore the prior-to-death disability profiles and their correlates among Chinese older adults. Specifically, three questions needed to be answered: (1) what are the disability profiles of Chinese older adults prior to death, (2) how are disability profiles linked to the end-of-life care arrangements and place of death of older adults, and (3) what are the predictors of disability profiles? Nationally representative data were used to guarantee the generalizability of the findings. Latent profile analyses, bivariate analysis, and multivariate logistic regression were applied to identify profiles, explore how disability profiles are linked with end-of-life care arrangements and place of death, and examine the effects of each predictor on disability profiles.

## Methods

### Design

The present study adopted a cross-sectional design, and data from the Chinese Longitudinal Healthy Longevity Survey (CLHLS) were analyzed. Details of the database have been reported by Yi [23]. Briefly, a nationally representative survey collected longitudinal data about adults aged 65 years and older in 22 out of 31 provinces across Mainland China. Basic information, financial status, health, function, ability, lifestyle, and care arrangements regarding the subjects were collected when they were alive. When a participant died, the next of kin was interviewed with an exit questionnaire on the conditions surrounding the death and dying of the older adult. The baseline survey was conducted in 1998, and follow-up with replacement for the deceased was performed in 2000, 2002, 2005, 2008, 2009, 2011, 2014, and 2018 [24]. In the present study, data were collected from exit questionnaires completed by deceased the older adult’s next of kin.

### Variables and measurements

Data relating to variables about the deceased older adult’s disabilities prior to death, end-of-life care arrangements, place of death, and potential predictors were extracted from the exit questionnaires.

In the CLHLS, disabilities were measured by the Activities of Daily Living (ADL) index [25]. Initially, designed in English, the tool was translated into Mandarin and successfully applied to Chinese older adults [26]. Six items asked the next of kin to recall the older adult’s abilities regarding bathing, dressing, toileting, indoor transferring, continence, and self-feeding prior to death. For each item except continence, higher scores represent less disability (0 = fully dependent, 1 = partially dependent, 2 = fully independent). For continence, a higher score represented stronger control (0 = totally incontinent of bowel or bladder, 1 = partially incontinent of bowel or bladder, 2 = complete self-control over urination and defecation).

End-of-life care arrangements included the older adult’s primary living arrangements prior to death (never-married and alone and widowed and alone are both recoded as “alone”; with married child (including grandchildren), with married grandchild (not including child), with unmarried child/grandchild, and with other relatives are all recoded as “with family or relatives”) and primary caregiver (children/their spouses, grandchildren/their spouses, other family members, and friends are all recoded as “family or friends”; social service providers and nurses/maids were both recoded as “professional caregivers”). The next of kin was asked to designate the place of death of the older adult as home, hospital, an institution, or other location.

Potential predictors included basic demographic information and health status [27, 28]. Demographics included the older adult’s age at death, year of death, sex, marital status, category of residence (urban/town/rural), insurance status, and yearly income of the family. Health status included the older adult’s medication and whether he or she had chronic diseases (hypertension, diabetes, heart disease, stroke or CVD, bronchitis/emphysema/pneumonia, tuberculosis, cancer, Parkinson’s disease, or dementia) prior to death.

### Participants

The present study targeted all deceased CLHLS participants whose next of kin completed the exit questionnaire in the 2011, 2014, or 2018 wave. A total of 10,681 cases were identified from the database. Among them, 5,642, 2,018, and 2,226 participants initially joined the CLHLS in the 2008 wave, the 2011 wave, and the 2014 wave, respectively. A total of 145 cases were excluded because all data on the 6 ADL items were missing, and another 7 cases were excluded because the reported age at death was less than 65 years. Eventually, data on 10,529 deceased participants were analyzed.

### Statistical analysis

After descriptive analyses were performed for all variables, LPA with unconditional models was run using the 6 ADL item scores as observed responses to identify the deceased participants’ disability patterns prior to death. Although there is usually more than one observed construct in LPA [29, 30], detailed analyses of single observed constructs such as depression [31] and social participation [32] have also been conducted with LPA among older adults. Models with 1 to 5 latent classes were examined, and missing data were addressed with maximum likelihood estimation. Model selection was based on statistical criteria and theoretical considerations. The statistical criteria included lower Akaike Information Criteria (AIC) and sample-size-adjusted Bayesian Information Criteria (aBIC), higher entropy (near 1), higher posterior probabilities (0.8 or larger), significant Lo-Mendell-Rubin Adjusted Likelihood Ratio test (LMR-LRT) and Bootstrapped Likelihood Ratio Test (BLRT) results [33], and the smallest class with a size of no less than 1% of the total sample.

For the selected model, the 6 ADL item scores in each class were calculated, and between-class differences were examined with ANOVAs and post hoc tests. Then, between-class comparisons of living arrangements prior to death, primary caregiver prior to death, and place of death were conducted with chi-square tests.

All potential predictors were then joined one by one in ANOVAs and chi-square tests to examine their relationship with latent class membership. Variables with significant bivariate links with latent class membership were joined together in the multinomial logistic regression to determine their predictive effects after shared variances were controlled. Dichotomous and categorical predictors were transferred to dummy variables before joining the regression. Missing data were addressed with multiple imputation in both bivariate analyses and regressions (number of imputations = 5) [34].

LPA was conducted with Mplus 7.4 [35], and all remaining analyses were performed with R 3.6.3 [36].

### Ethical concerns

Since no new data were collected, no ethical approval was needed for the present study. CLHLS was approved by the research ethics committees of Peking University (reference number: IRB00001052-13074) and Duke University (reference number: Pro00062871), and all participants or their next of kin (when participants could not write) provided written consent before joining the study.

## Results

### Basic information and prior-to-death status of participants

The final sample consisted of 4,266 males (40.5%) and 6263 females (59.5%), and the mean age at death was 94.56 (65–121, SD = 8.95). The mean total ADL score was 4.87 (*n* = 10,136, *SD* = 4.48), while the functional scores for bathing, dressing, toileting, indoor transferring, continence, and self-feeding were 0.62 (*n* = 10,475, *SD* = 0.80), 0.74 (*n* = 10,475, *SD* = 0.86), 0.71 (*n* = 10,508, *SD* = 0.85), 0.69 (*n* = 10,485, *SD* = 0.85), 1.23 (*n* = 10,477, *SD* = 0.87), and 0.90 (*n* = 10,481, *SD* = 0.88), respectively. Information on the 10,529 participants is shown in Table 1.

**Table 1:** Basic information and prior-to-death status of the 10,529 participants

Variable	*N*	M (SD)/%	Variable	*N*	M (SD)/%
Sex	10,529		Yearly income per capita of the family	10,304	
Male	4,266	40.5	$<$10,000	3,662	35.5
Female	6,263	59.5	10,000–49,999	4,369	42.4
Age	10,529	94.56 (8.954)	$\ge$50,000	2,273	22.1
Marital status	10,514		Have public old-age insurance	10,349	
Married and living with the spouse	1,849	17.6	Yes	2,259	21.8
Married but not living with the spouse	210	2.0	No	8,090	78.2
Divorced	17	0.2	Suffered from diseases		
Widowed	8,339	79.3	Hypertension	10,251	
Never married	99	0.9	No	8,039	78.7
Category of residence	10,515		Yes	2,176	21.3
City	1,465	13.9	Diabetes	10,170	
Town	2,646	25.2	No	9,759	96.0
Rural	6,404	60.9	Yes	411	4.0
Validated year of death	10,529		Heart disease	10,215	
2008	747	7.1	No	8,658	84.8
2009	1,889	17.9	Yes	1,557	15.2
2010	1,825	17.3	Stroke or CVD	10,202	
2011	1,280	12.2	No	8,847	86.7
2012	1,122	10.7	Yes	1,355	13.3
2013	1,043	9.9	Bronchitis, emphysema, pneumonia	10,186	
2014	786	7.5	No	8,719	85.6
2015	625	5.9	Yes	1,467	14.4
2016	570	5.4	Tuberculosis	10,169	
2017	458	4.3	No	10,060	98.9
2018	170	1.6	Yes	109	1.1
2019	14	0.1	Cancer	10,176	
Primary living arrangement prior to death	9,038		No	9,643	94.8
Nursing home	239	2.6	Yes	533	5.2
Alone	1,024	11.3	Parkinson’s disease	10,166	
With old spouse only	1,318	14.6	No	10,076	99.1
With family or relatives	6,323	70.0	Yes	90	0.9
Other	134	1.5	Dementia	10,183	
Primary caregiver prior to death	10,514		No	9,583	94.1
Spouse	802	7.6	Yes	600	5.9
Family and friends	8,910	84.7	Got timely medication	7,682	
Professional caregivers	470	4.5	Yes	5,828	75.9
Nobody	70	0.7	No	324	4.2
Do not need care	262	2.5	Was not sick	1,530	19.9
Place of death	10,514				
Home	9,383	89.2			
Hospital	830	7.9			
Institutions	242	2.3			
Other	59	0.6			

### Latent ADL patterns

The parameters of the models with 1 to 5 latent classes are shown in Table 1 of the Appendix [see Additional file 1]. The 3-class model was selected because it had the lowest AIC (92,861) and aBIC (92,968) and satisfactory entropy (0.952). Furthermore, both LMR-LRT and BLRT showed that the 3-class model suited the data better than did the 1-class (*ps*$<$ 0.001) and 2-class (*ps*$<$ 0.001) models and equally to the 4-class (*ps*$>$ 0.05) and 5-class (*ps*$>$ 0.05) models.

**Fig. 1:**
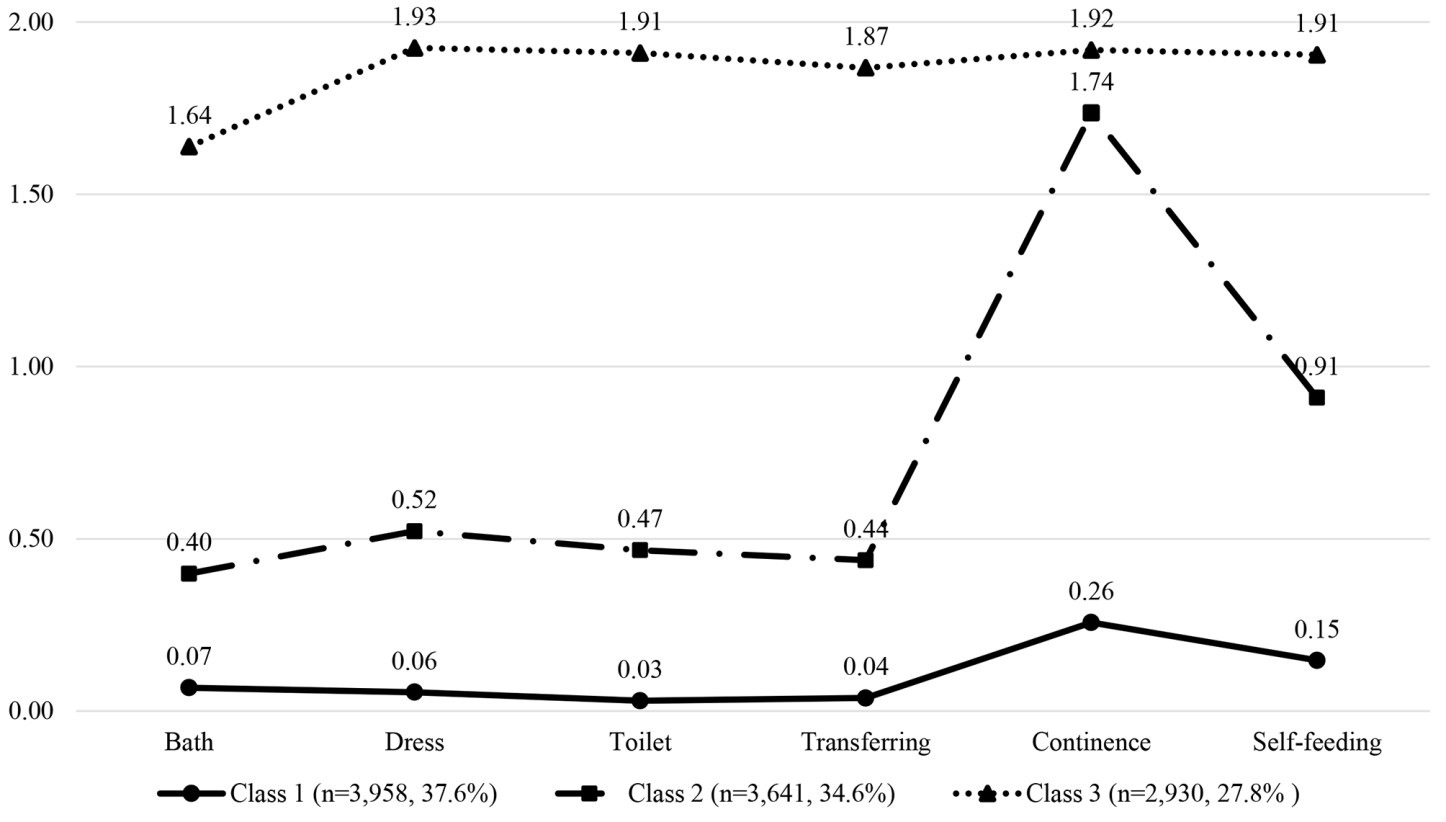
Disability patterns of the 10,529 participants prior to death

According to the three-class model, 3,958, 3,641, and 2,930 participants were in Class 1 (37.6%), Class 2 (34.6%), and Class 3 (27.8%), respectively. Figure 1 shows the disability profiles of the three classes. Between-class comparisons of total ADL scores and item scores are shown in Table 2 of the Appendix [see Additional file 2]. For the total ADL score and each item score, participants in Class 1 scored significantly lower than those in Class 2, and the scores of Class 2 were significantly lower than those in Class 3. In addition, Class 2 had a relatively high score for continence. Therefore, Class 1, Class 2, and Class 3 were named “Disabled-Incontinent”, “Disabled-Continent”, and “Independent”, respectively.

### End-of-life care arrangements and place of death

Table 2 shows the between-class differences in end-of-life care arrangements and the place of death. The three groups were significantly different in primary living arrangement prior to death (${\chi }^{2}$ = 32.550, *p*$<$ 0.001), primary caregiver prior to death (${\chi }^{2}$ = 32.550, *p*$<$ 0.001), and place of death (${\chi }^{2}$ = 32.550, *p*$<$ 0.001). In general, older adults in the Independent group were more likely to live alone or with a spouse before death, and they were more likely to receive no care or care only from their spouse. Regarding place of death, while older adults in the Disabled-Continent group had a higher chance of dying at home, those in the other two groups were more likely to die in hospitals or institutions.

**Table 2:** Between-class differences in end-of-life care arrangements and place of death

Variable	Disabled-Incontinent	Disabled-Continent	Independent	${\varvec{\chi }}^{2}$
Primary living arrangement prior to death (*n* = 9038)				32.55^***^
Nursing home				
Living arrangement, %	52.3	24.3	23.4	
Class, %	3.7	1.9	2.2	
Alone				
Living arrangement, %	35.3	34.4	30.4	
Class, %	10.7	11.2	12.2	
With old spouse only				
Living arrangement, %	35.5	34.1	30.4	
Class, %	13.9	14.3	15.8	
With family or relatives				
Living arrangement, %	37.3	35.3	27.4	
Class, %	70.1	71.2	68.3	
Other				
Living arrangement, %	39.6%	33.6%	26.9%	
Class, %	1.6	1.4	1.4	
Primary caregiver prior to death (*n* = 10,514)				600.83^***^
Spouse				
Living arrangement, %	34.0	33.5	32.4	
Class, %	6.9	7.4	8.9	
Family or friends				
Living arrangement, %	38.4	35.8	25.8	
Class, %	86.6	87.7	78.7	
Professional caregivers				
Living arrangement, %	49.1	33.2	17.7	
Class, %	5.8	4.3	2.8	
Nobody				
Living arrangement, %	17.1	8.6	74.3	
Class, %	0.3	0.2	1.8	
Do not need care				
Living arrangement, %	5.7	6.9	87.4	
Class, %	0.4	0.5	7.8	
Place of death (*n* = 10,514)				14.48^***^
Home				
Living arrangement, %	37.4	35.2	27.4	
Class, %	88.8	90.9	87.8	
Hospital				
Living arrangement, %	36.0	32.4	31.6	
Class, %	7.6	7.4	9.0	
Institutions				
Living arrangement, %	53.3	24.0	22.7	
Class, %	3.3	1.6	1.8	
Other				
Living arrangement, %	23.7	6.8	69.5	
Class, %	0.4	0.1	1.4	

a *p < .05; **p < .01; ***p < .001.

### Predictive factors of latent class membership

The outcomes of the bivariate analyses between latent class membership and each potential predictor are shown in Table 3 of the Appendix [see Additional file 3]. Except for the family’s yearly income, category of residence, and presence of Parkinson’s disease, all other predictors had a significant one-to-one relationship with latent class membership.

The findings of the multinomial logistic regression with all significant predictors in bivariate analyses are listed in Table 3. In general, being female, not “married and living with the spouse”, suffering from hypertension, diabetes, stroke or CVD, bronchitis/emphysema/pneumonia, cancer, or dementia, and dying at a later year were linked to more severe disability patterns prior to death. Moreover, not having public old-age insurance predicted a lower likelihood of being in the Disabled-Incontinent group before death, while the chance of being in the Disabled-Continent group before death increased with age.

**Table 3:** Outcomes of multinomial logistic regression

Variable	Disabled-Continent vs. Disabled-Incontinent ^a^	Independent vs. Disabled-Incontinent	Independent vs. Disabled-Continent
Age	1.01^**^	0.98^***^	0.97^***^
Sex (ref = male)			
Female	0.94	0.75^***^	0.80^***^
Marital status (ref = Married and living with the spouse)			
Married but not living with the spouse	0.57^***^	0.83^**^	1.45^***^
Divorce	1.02	1.53	1.50^*^
Widowed	0.87^***^	0.83^***^	0.96
Never married	0.95^**^	0.89^**^	0.94^*^
Validated year of death	0.99^***^	0.98^***^	0.99^***^
Have public old-age insurance (ref = yes)			
No	1.12^**^	1.12^**^	0.99
Suffer from disease (ref = no)			
Hypertension	0.98	0.87^**^	0.89^**^
Diabetes	0.92^**^	0.82^*^	0.89
Heart disease	0.94	1.04	1.11^*^
Stroke or CVD	0.52^***^	0.29^***^	0.55^***^
Bronchitis/emphysema/pneumonia	1.08	0.85^***^	0.79^***^
Tuberculosis	0.67	1.10	1.65
Cancer	1.06	0.55^***^	0.52^***^
Dementia	0.44^***^	0.30^***^	0.70^***^
Got timely medication (ref = yes)			
No	0.87	0.54	0.63^*^
Was not sick	1.22^*^	1.89^***^	1.55^***^

a *p < .05; **p < .01; ***p < .001.

## Discussion

### Prior-to-death disability profiles among the Chinese older adults

With data from 10,529 Chinese older adults who died between 2008 and 2019, the present study identified three prior-to-death disability patterns associated with LPA: disabled-incontinent (37.6%), disabled-continent (34.6%), and independent (27.8%). The proportion of individuals with disability was higher than that reported by Liu, Han [5], who reported that approximately 48% of Chinese older adults in the CLHLS who died between 1998 and 2014 had ADL disability 1 month prior to death. This difference can be attributed to the development of medical and social services between 1998 and 2014 and between 2008 and 2019. With help from medication and professional caregivers, the survival of older adults unable to perform ADLs can be prolonged. This is consistent with the findings of the present study, in which older adults who died at a later year were more likely to have a more severe disability profile. In general, these profiles show that the need for disability support before death among Chinese older adults was quite high since nearly 3/4 of them experienced functional disability prior to death. Functional disability should be considered the status quo and a challenging issue by policy makers, service designers, researchers, educators and frontline workers in the field of end-of-life care.

Incontinence is a key indicator that distinguishes between two disability profiles among Chinese older adults at the end of life, and those with incontinence also have more severe functional disability in terms of other ADLs. According to a previous analysis [37], the oldest adults generally lose the ability to perform ADLs in the order of bathing, toileting, transferring, dressing, eating, and continence, and incontinence is the only aspect of ADLs that cannot benefit from either physical leisure activities or balanced leisure activities among older adults [38]. Therefore, incontinence may signify severe illness and even terminal stage in the dying process. Incontinence deprives dying patients of their sense of control [39] and poses an enormous and confusing challenge for caregivers [40]. Addressing incontinence would help maintain the patient’s dignity and quality of life in the last days [41]. However, except for the finding that calf circumference measurements below a certain threshold indicate a risk of incontinence in the elderly Chinese population [42], there are few reports on the preferences of dying patients regarding the management of incontinence-related symptoms [43] or evidence-based suggestions for managing incontinence at the end of life [44]. It is necessary to look more closely into the needs of dying patients who are incontinent and the more complicated disability profile accompanying such a symptom to enable more adapted services.

### End-of-life care arrangements and place of death

In the present study, we can see that end-of-life care arrangements for older adults changed with the disability profile. Independent older adults were more likely to live with and be cared for by spouses and other family members and were less likely to seek help from professional services. Nevertheless, it is worth noting that the majority of older adults, regardless of their disability profiles, received care in the family setting prior to death. This can be attributed to both the relatively slow development of institutional palliative care in mainland China [45] and the strong influence of filial piety values in the country [18]. Under such circumstances, helping family caregivers provide tailored end-of-life care to older adults with various disability profiles in the family-care setting, raising awareness among families about when institutional care would be necessary, and guaranteeing familial involvement even in cases of institutional care are of paramount importance in the Chinese context.

Most of the Chinese older adults in the present study eventually died at home. Moreover, the proportion of home deaths was slightly higher among individuals with the Disability-Continence profile than among those with the Disability-Incontinence profile or the Independent profile. Independent older adults are unlikely to die from chronic conditions, so they are more likely to be sent to hospitals due to acute situations and die there. Meanwhile, the home care of older adults with the Disability-Incontinence profile may be too challenging for the family in the last few days. Therefore, they must be sent to hospitals and institutions for help.

### Predictors of ADL patterns

After age at death, year of death, marital status, access to old-age insurance, and chronic diseases were controlled, females were found to have a higher likelihood than males of having a Disabled-Incontinence profile rather than a Disabled-Continence or Independent profile. Similarly, a study with 4,875 community-dwelling Japanese decedents aged 65 years and older also revealed that women had more severe disability than men in the last month of life [11]. The difference can be explained by sex roles: the older adults in the present study (the youngest were born in 1953) were born before the reform and opening up of China in 1978. According to the study by Luo [46], this cohort is relatively less egalitarian in terms of gender attitudes such as “men should be career oriented while women family oriented” than those born after 1978. When older women are expected to be and are actually playing the role of major family caregiver, their perceived social support and instrumental social support are lower than those of men [47], and the limited care they receive could lead to more severe disability or hastened progression to disability during their last days.

Regardless of sex, being married and living with the spouse are related to less problematic disability profiles. Compared with older adults who were married and living with the spouse, those who were married but not living with the spouse, widowed, or never married had poorer mental health [48], which can increase the risk of functional disability in this age group [49].

In the present study, hypertension, diabetes, stroke or CVD, bronchitis/emphysema/pneumonia, cancer, and dementia all predicted a more severe disability profile in an older Chinese adult prior to death. Associations between chronic diseases and disability were also found among Brazilian [50] and Dutch [51] older adults. More instructions and support need to be given to older patients with chronic diseases and their families to prepare them for the dying process with higher risks of disability.

With increasing age, the chances of an older adults dying without any disability decrease. However, in the present study, those who died at an advanced age were more likely to have the Disabled-Continent rather than the Disabled-Incontinent profile. Those who died at very old ages may be more likely to die from natural causes and be in a fragile rather than severely ill state at the end of life. Thus, more of them do not suffer from incontinence or severe disability.

Older adults who do not have public old-age insurance are more likely to have an Independent or Disabled-Continent profile than a Disabled-Incontinent profile. China is striving to develop a pension system featuring universal coverage, although the system is currently weak and incomplete [52]. Without support from old-age insurance, older adults are more likely to die from diseases that require unaffordable medical resources when they are still quite independent or have only the Disabled-Continent profile before they deteriorate into the Disabled-Incontinent state.

### Dying disabled and incontinent: Pain or privilege?

In the present study, different predictors of the Disabled-Incontinent profile seem to tell contrasting stories. On the one hand, unfavorable factors such as a series of chronic diseases, less access to social support when growing old, and not remaining “married and living with a spouse” before death predicted a higher likelihood of suffering from the Disabled-Incontinent profile. On the other hand, favorable factors such as dying in a later year with better developed medical and social services and support from old-age insurance can also contribute to dying with the Disabled-Incontinent profile. It is possible that while certain risk factors threaten older adults’ independence prior to death, only under favorable conditions can older adults survive moderately challenging situations and not die until they are entirely disabled and incontinent.

### Contributions and limitations

The present study is the first to focus on heterogeneous disability patterns in older adults prior to death. Previous studies on the disability patterns of older adults’ either focused on advanced age in general rather than the near-death period [27] or examined changing trajectories approaching death rather than distribution profiles prior to death [16]. Moreover, the present study is one of the first to discuss disability among older adults’ in light of end-of-life care. Three latent disability profiles were identified among a nationally representative sample, and these disability profiles were found to be linked to the end-of-life care arrangements and place of death of older adults. Moreover, both basic demographics and health conditions significantly predicted latent class membership, and dying disabled and incontinent individuals were characterized by both risk factors and favorable conditions. These findings elucidate the role of disability in the dying process, end-of-life care arrangements, and outcomes of Chinese older adults, as well as their shaping forces. They also lay the foundation for researchers, policy makers, and frontline practitioners to develop tailored support plans for both dying patients and their families. In addition, the framework and analysis can inspire explorations in other parts of the world.

Two limitations need to be considered when interpreting the present findings. First, the reports given by the next of kin regarding the condition of the older adults before death may not be entirely accurate, so bias might have been introduced. Second, owing to the secondary analysis design, while certain variable categories were considered for theoretical reasons, the selection of specific variables within each category was limited to those available in the CLHLS database. Consequently, additional variables and measurements that could have further served the research objectives were not included in the present analysis.

### Practical implications

In the Chinese setting, support for functional disability is important since nearly 3/4 of Chinese older adults suffer from disabilities prior to death, and this proportion may increase with the development of medical, care, and pension systems in China. More effort is needed to support home-based end-of-life caregiving and fulfill the wishes of older adults for a home death. However, hospitals and long-term care institutions should also prepare for complicated end-of-life cases. More serious attention should be given to older adults who are female, not “married and living with the spouse”, and who have chronic diseases such as hypertension, diabetes, stroke or CVD, bronchitis/emphysema/pneumonia, cancer, or dementia due to their high risk of developing the Disabled-Incontinent profile prior to death. For these individuals, caring for incontinence is imperative to improve quality of life and maintain dignity.

### Future studies

Future studies can involve more predictors at the social level for disability in older adults prior to death, and cross-cultural comparisons can be made to understand the influence of cultural and systemic forces. More in-depth explorations should be conducted to understand the needs of patients and their family caregivers in the face of different prior-to-death disability profiles. On such a basis, more tailored end-of-life care policy and intervention plans can be developed and tested.

## Conclusions

Among Chinese adults aged 65 years and older, three prior-to-death disability patterns, namely, Disabled-Incontinent (37.6%), Disabled-Continent (34.6%), and Independent (27.8%), were found. These profiles share significant links with the end-of-life care arrangements of older adults and their place of death. Both demographic information and health status can predict prior-to-death disability profiles. Dying after becoming disabled and incontinent occurred among individuals who experienced certain risk factors as well as favorable conditions.

### Electronic supplementary material

Below is the link to the electronic supplementary material.

Supplementary Material 1

Supplementary Material 2

Supplementary Material 3

## Data Availability

The data that support the findings of this study are available from the corresponding author, Dr. Chuqian Chen (email: ccq0213@outlook.com.), upon reasonable request.

## Abbreviations

CLHLS: The China Longitudinal Survey on Health and Longevity.
CVD: Cerebrovascular disease.
LPA: Latent profile analysis.
LCGA: Latent classic growth analysis.
BMI: Body mass index.
ADL: Activities of Daily Living.
