# Supplementary material for: Chinese older adults’ prior-to-death disability profiles and their correlates
Source: BMC Geriatr. 2024 Jun 1;24:479. doi: 10.1186/s12877-024-05105-y (PMC11143689; doi:10.1186/s12877-024-05105-y)
Supplement: Supplementary file 1 — Supplementary Material 1 [file 12877_2024_5105_MOESM1_ESM.docx]

Additional file 1

Supplementary Table 1. Outcomes of models with 1 to 5 latent classes.

| N  of  classes | AIC^a^ | aBIC^a^ | C_1_^a^ | C_2_ | C_3_ | C_4_ | C_5_ | entropy | posterior probabilities | LMR-LRT^a,b^ | BLRT^a,b^ |
| --- | --- | --- | --- | --- | --- | --- | --- | --- | --- | --- | --- |
| 1 | 158915 | 158364 | 10529 |  |  |  |  |  |  |  |  |
| 2 | 102496 | 102573 | 6460 | 4069 |  |  |  | .970 | .992,  .992 | *** | *** |
| 3 | 92861 | 92968 | 3958 | 3641 | 2930 |  |  | .952 | .979,  .974,  .993 | *** | *** |
| 4 | 92875 | 93010 | 0 | 3641 | 3958 | 2930 |  | .962 | 0, .974,  .979,  .993 |  |  |
| 5 | 92889 | 93053 | 0 | 0 | 3958 | 3641 | 2930 | .919 | 0, 0,  .937,  .974,  .993 |  |  |
| aAIC: Akaike Information Criteria; aBIC: sample-size-adjusted Bayesian Information Criteria; C_n_: size of the n^th^ group; LMR-LRT: Lo-Mendell-Rubin Adjusted LRT test; BLRT: Bootstrapped Likelihood Ratio Test.  b *p < .05; **p < .01; ***p < .001. | | | | | | | | | | | |
