# Supplementary material for: Chinese older adults’ prior-to-death disability profiles and their correlates
Source: BMC Geriatr. 2024 Jun 1;24:479. doi: 10.1186/s12877-024-05105-y (PMC11143689; doi:10.1186/s12877-024-05105-y)
Supplement: Supplementary file 2 — Supplementary Material 2 [file 12877_2024_5105_MOESM2_ESM.docx]

Additional file 2

Supplementary Table 2. Between-class comparisons of ADL item scores and total scores.

| ADL |  | *M* (*SD*) |  | F^a^ | Post-hoc tests^a^ | | |
| --- | --- | --- | --- | --- | --- | --- | --- |
|  | Class 1 | Class 2 | Class 3 |  | Class 1 V Class 2 | Class 1 V Class 3 | Class 2 V Class 3 |
| Bath | .07 (.26) | .40 (.52) | 1.64 (.60) | 9981.94^***^ | *** | *** | *** |
| Dress | .06 (25) | .52 (.57) | 1.93 (.28) | 19355.99^***^ | *** | *** | *** |
| Toilet | .03 (.17) | .47 (.52) | 1.91 (.30) | 24339.33^***^ | *** | *** | *** |
| Transferring | .04 (.20) | .44 (.53) | 1.87 (.36) | 20294.77^***^ | *** | *** | *** |
| Continence | .26 (.44) | 1.74 (.47) | 1.92 (.31) | 17365.44^***^ | *** | *** | *** |
| Self-feeding | .15 (.40) | .91 (.74) | 1.91 (.35) | 9079.00^***^ | *** | *** | *** |
| Total score | .58 (.86) | 4.49 (1.97) | 11.16 (1.31) | 44252.29^***^ | *** | *** | *** |
| a *p < .05; **p < .01; ***p < .001. | | | | | | | |
