# Supplementary material for: Chinese older adults’ prior-to-death disability profiles and their correlates
Source: BMC Geriatr. 2024 Jun 1;24:479. doi: 10.1186/s12877-024-05105-y (PMC11143689; doi:10.1186/s12877-024-05105-y)
Supplement: Supplementary file 3 — Supplementary Material 3 [file 12877_2024_5105_MOESM3_ESM.docx]

Additional file 3

Supplementary Table 3. Outcomes of bivariate analyses.

| Variable | *M*(*SD*) / % | | | F/ $\boldsymbol{\chi}^{\boldsymbol{2}}$^a^ |
| --- | --- | --- | --- | --- |
|  | Disabled-Incontinent | Disabled-Continent | Independent |  |
| Age | 94.31 (9.02) | 95.38 (8.70) | 93.87 (9.11) | 25.54^**^ |
| Sex |  |  |  | 22.63^***^ |
| Male | 38.5 | 38.5 | 45.7 |  |
| Female | 61.5 | 61.5 | 54.3 |  |
| Marital status |  |  |  | 3.19^**^ |
| Married living with the spouse | 16.7 | 16.8 | 19.8 |  |
| Married but not living with the spouse | 2.3 | 1.5 | 2.2 |  |
| Divorce | .1 | .1 | .2 |  |
| Widowed | 80.0 | 80.7 | 76.7 |  |
| Never married | .8 | .9 | 1.1 |  |
| Validated year of death |  |  |  | 6.13^**^ |
| 2008 | 6.4 | 7.1 | 7.9 |  |
| 2009 | 17.0 | 18.7 | 18.3 |  |
| 2010 | 16.5 | 17.5 | 18.2 |  |
| 2011 | 12.6 | 12.2 | 11.6 |  |
| 2012 | 10.8 | 10.9 | 10.2 |  |
| 2013 | 10.6 | 9.3 | 9.7 |  |
| 2014 | 8.3 | 6.7 | 7.3 |  |
| 2015 | 5.9 | 6.3 | 5.6 |  |
| 2016 | 5.4 | 5.4 | 5.5 |  |
| 2017 | 4.4 | 4.2 | 4.4 |  |
| 2018 | 1.9 | 1.5 | 1.3 |  |
| 2019 | .2 | .1 | .1 |  |
| Yearly income per capita of the family |  |  |  | .51 |
| $\boldsymbol{<}$10000 | 35.0 | 35.8 | 35.9 |  |
| 10000-49999 | 42.4 | 42.9 | 41.9 |  |
| $\boldsymbol{\geq}$50000 | 22.6 | 21.3 | 22.2 |  |
| Category of residence |  |  |  | 1.51 |
| City | 14.6 | 13.1 | 14.0 |  |
| Town | 25.8 | 25.0 | 24.5 |  |
| Rural | 59.6 | 61.9 | 61.5 |  |
| Have public old-age insurance | 23.6 | 20.5 | 21.0 | 6.36^**^ |
| Got timely medication |  |  |  | 39.32^***^ |
| Yes | 79.6 | 76.5 | 70.2 |  |
| No | 5.4 | 4.4 | 2.4 |  |
| Was not sick | 14.9 | 19.0 | 27.4 |  |
| Hypertension | 25.0 | 20.1 | 17.8 | 29.03^***^ |
| Diabetes | 5.1 | 3.6 | 3.2 | 8.21^***^ |
| Heart disease | 16.9 | 14.1 | 14.5 | 6.29^**^ |
| Stroke or cvd | 20.5 | 10.8 | 6.6 | 158.46^***^ |
| Bronchitis, emphysema, pneumonia | 15.2 | 14.8 | 12.9 | 3.58^*^ |
| Tuberculosis | 1.3 | .7 | 1.2 | 3.17^*^ |
| Cancer | 5.7 | 5.8 | 4.0 | 6.35^**^ |
| Parkinson's disease | 1.2 | .8 | .6 | 3.05 |
| Dementia | 9.9 | 4.1 | 2.8 | 92.20^***^ |
| a *p < .05; **p < .01; ***p < .001. | | | | |
